# Successful treatment of severe adrenaline-resistant anaphylactic shock with glucagon in a patient taking a beta-blocker: a case report

**DOI:** 10.1186/s40981-021-00490-4

**Published:** 2021-12-15

**Authors:** Yu Murakami, Shohei Kaneko, Haruka Yokoyama, Hironori Ishizaki, Motohiro Sekino, Hiroaki Murata, Tetsuya Hara

**Affiliations:** Department of Anesthesiology and Intensive Care Medicine, Nagasaki University Graduate School of Biomedical Sciences, 1-7-1 Sakamoto, Nagasaki, 852-8501 Japan

**Keywords:** Anaphylaxis, Therapeutic management, Adrenaline, β-blockers, Glucagon

## Abstract

**Background:**

The efficacy of glucagon for adrenaline-resistant anaphylactic shock in patients taking β-blockers is controversial. However, understanding the efficacy of glucagon is important because adrenaline-resistant anaphylactic shock is fatal. We present a case of severe adrenaline-resistant anaphylactic shock in a patient taking a β-blocker, and glucagon was effective in improving hemodynamics.

**Case presentation:**

An 88-year-old woman with severe aortic stenosis and taking a selective β-1 blocker underwent transcatheter aortic valve implantation under general anesthesia. Postoperatively, she received 100 mg sugammadex, but 2 min later developed severe hypotension and bronchospasm. Suspecting anaphylactic shock, we intervened by administering adrenaline, fluid loading, and an increased noradrenaline dose. Consequently, the bronchospasm improved, but her blood pressure only increased minimally. Therefore, we administered 1 mg glucagon intravenously, and the hypotension resolved immediately.

**Conclusions:**

Glucagon may improve hemodynamics in adrenaline-resistant anaphylactic shock patients taking β-blockers; however, its efficacy must be further evaluated in more cases.

## Background

Although adrenaline is the first-line treatment for anaphylactic shock, its efficacy may be limited in patients taking β-blockers regularly [1]. Glucagon exerts positive inotropic and chronotropic effects by directly activating adenylyl cyclase and bypassing β-adrenergic receptor blockade [2]. Therefore, glucagon may be effective in improving hemodynamics in patients taking β-blockers regularly [3, 4]. International guidelines and consensus also recommended glucagon administration for adrenaline-resistant anaphylactic shock in such patients [5–9]. However, some experts suggest re-evaluating this recommendation [10], because the evidence supporting glucagon administration is based on only two case reports from two to three decades ago [3, 4]. Understanding the role and efficacy of glucagon is important because adrenaline-resistant anaphylactic shock is fatal [11]. Nevertheless, few reports have demonstrated the efficacy of glucagon for refractory anaphylactic shock.

Herein, we present a case of severe adrenaline-resistant anaphylactic shock caused by sugammadex following transcatheter aortic valve implantation (TAVI) under general anesthesia in a patient taking a β-blocker regularly, and glucagon was effective in improving hemodynamics.

### Case presentation

An 88-year-old woman (height, 150 cm; weight, 41 kg) with no history of drug allergy or general anesthesia was scheduled for transfemoral TAVI because of severe aortic stenosis (AS). The surgery was performed under general anesthesia to facilitate the use of transesophageal echocardiography and to manage any intraoperative complications. She was taking 0.625 mg/day bisoprolol, a selective β-1 blocker, for hypertension and chronic atrial fibrillation. Additionally, she was taking amlodipine for hypertension. Preoperative transthoracic echocardiography showed severe AS (aortic valve peak flow velocity: 4.73 m/s; mean aortic valve pressure gradient: 47 mmHg; aortic valve area: 0.41 cm^2^) and myocardial hypertrophy (left ventricular posterior wall thickness and interventricular septum thickness: 14 mm).

**Fig. 1:**
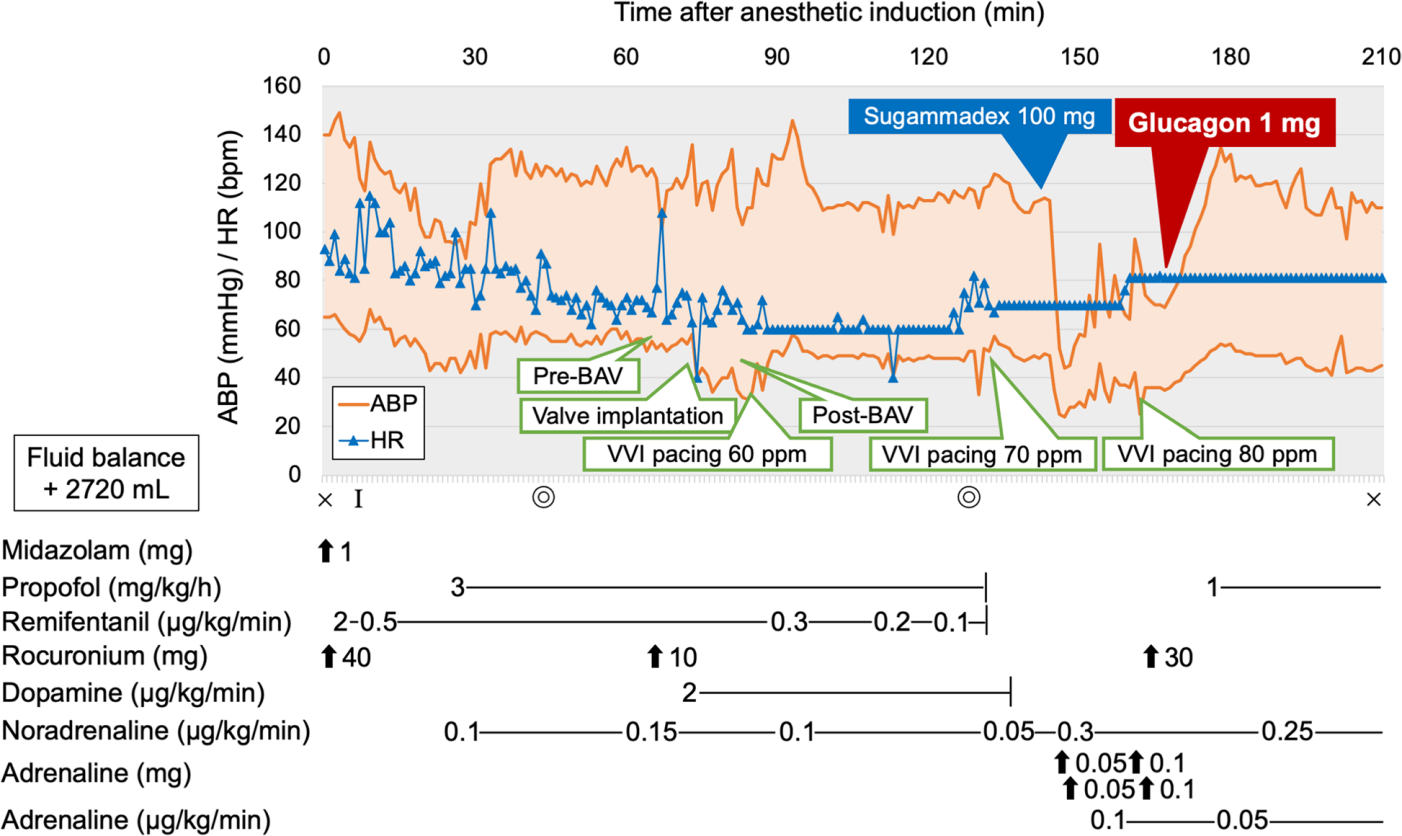
Anesthesia record. × Anesthesia start and end. I intubation. Concentric circles: Surgery start and end. ABP = arterial blood pressure; bpm = beats per minute; BAV = balloon aortic valvuloplasty; HR = heart rate; ppm = pulses per minute

Pre-anesthetic medication for sedation was not administered. She received bisoprolol and amlodipine 3 h before entering the operating room. Figure 1 shows the anesthesia record. General anesthesia was induced using midazolam and remifentanil; additionally, rocuronium was administered to facilitate tracheal intubation. She received total intravenous anesthesia: continuous infusions of propofol and remifentanil. Noradrenaline and dopamine were infused continuously through the central venous catheter to maintain blood pressure. AS disappeared after valve implantation. During the surgery, complete atrioventricular block occurred, and ventricular pacing (VVI mode: 60 ppm) was initiated. No other complications were associated with the surgical procedure. Postoperatively, she received 100 mg sugammadex through the central venous catheter, but 2 min later, her systolic arterial blood pressure (ABP) decreased unexpectedly to less than 40 mmHg. Simultaneously, ventilator monitoring revealed elevated peak and plateau airway pressures (60 and 40 cmH_2_O, respectively). No skin rash was observed on the body surface. Transthoracic echocardiography showed underfilling of the left ventricle without right ventricular dilatation or pericardial effusion. No abnormalities were observed in left ventricular wall motion.

The patient developed distributive shock, which was clinically diagnosed as anaphylactic shock caused by sugammadex because of the rapid onset of severe hypotension and bronchospasm. First, two boluses of 0.05 mg adrenaline were administered through the central venous catheter, followed by rapid volume resuscitation using crystalloid (1 L infusion within 30 min). The dose of noradrenaline was increased to 0.3 μg/kg/min. Consequently, the airway pressures returned to the original level, but the hypotension persisted. Thereafter, adrenaline was administered via bolus (0.1 mg twice) and continuous infusion (0.1 μg/kg/min); however, the increase in her ABP was minimal. Therefore, 1 mg glucagon was administered intravenously, and her systolic ABP immediately rose to 130 mmHg. An erythematous rash also appeared on her neck with the increase in ABP. Her general condition was stable after the therapeutic interventions. Ventricular pacing was continued because bradycardia with complete atrioventricular block was persistent during the interventions. She was admitted to the intensive care unit under tracheal intubation. Subsequently, the continuous infusion of adrenaline was discontinued, and the continuous infusion of noradrenaline was reduced. We also administered 125 mg methylprednisolone for 3 days. She was extubated 9 h after the onset of anaphylaxis, and circulatory agonists were discontinued the following day. She was discharged without complications on postoperative day 9. Blood tests revealed a total serum tryptase level of 7.3 ng/mL and 1.2 ng/mL at 1 h and 24 h after onset, respectively. We advised the patient to undergo allergy tests, such as skin prick tests or intradermal tests, to identify the cause of anaphylaxis. However, the patient refused these tests because she was elderly and unlikely to undergo surgery under general anesthesia in the future.

Written informed consent was obtained from the patient for the publication of this case report. This report was approved by the Institutional Review Board of Nagasaki University Hospital (Approval number: 21041932).

## Discussion

Adrenaline is the first-line treatment for any type of anaphylaxis [5–8], and it treats hypotension by increasing peripheral resistance via α-1 receptors and increasing cardiac output via β-1 receptors. Its β-2 adrenergic activity can reverse bronchospasm and treat lower respiratory tract symptoms. Additionally, by activating β-2 receptors on mast cells and basophils, adrenaline decreases the release of additional inflammatory mediators. However, patients taking β-blockers regularly are more prone to severe anaphylaxis because of the reduced effect of adrenaline [1]. In our patient, severe distributive shock occurred immediately after sugammadex administration. The patient was an elderly woman taking a selective β-1 blocker regularly who underwent treatment for AS. Despite suspecting anaphylactic shock early on and administering adrenaline, refractory hypotension persisted. However, a single dose of 1 mg glucagon markedly improved hemodynamics.

Glucagon is a peptide hormone physiologically produced by the α cells of the pancreas, and it exerts positive inotropic and chronotropic effects by directly activating adenylyl cyclase and bypassing β-adrenergic receptor blockade [2]. International guidelines and consensus recommended glucagon administration to improve hemodynamics for adrenaline-resistant anaphylactic shock in patients taking β-blockers regularly [5–9], and the initial recommended bolus dose of glucagon is 1–2 mg intravenously [6]. On the other hand, some experts suggest re-evaluating this recommendation for glucagon administration [10], because the evidence supporting glucagon administration is based on only two case reports from two to three decades ago [3, 4]. They reviewed these two case reports and pointed out that the adrenaline dose before glucagon administration was lower than the recommended dose in one case and higher than the recommended dose in the other case, and therefore, hemodynamic improvement could have been achieved without glucagon if the appropriate adrenaline dose had been administered [10]. Thus, to rigorously confirm the efficacy of glucagon for refractory anaphylactic shock, it must only be administered and its effect on hemodynamics assessed after the appropriate dose of adrenaline, according to the treatment flowchart for anaphylactic shock provided by international guidelines, is administered. However, to the best of our knowledge, no reports to date have documented such an analysis of the effects of glucagon in patients with refractory anaphylactic shock.

According to the practical guidelines for the response to perioperative anaphylaxis provided by the Japanese Society of Anesthesiologists, the recommended intravenous bolus dose of adrenaline for anaphylactic shock collapsed circulation is 0.05–0.3 mg [9]. Furthermore, another international consensus states that if shock persists, continuous infusion of adrenaline (0.05–0.1 μg/kg/min) should be given. Meanwhile, adrenaline can decrease coronary blood flow through coronary artery vasoconstriction, increase myocardial oxygen demand, and exacerbate myocardial ischemia [12]. Accordingly, adrenaline administration may increase the risk of myocardial ischemia in patients with coronary artery stenosis or myocardial hypertrophy, and hence, it should be administered cautiously even during anaphylaxis treatment [13]. In our patient, adrenaline was administered in parallel as bolus and continuous infusion. Considering the patient’s myocardial hypertrophy, we administered adrenaline cautiously to avoid any overdose, and the dose was within the recommended range suggested by the international guidelines and consensus. Additionally, adequate fluid loading and continuous infusion of noradrenaline were performed; however, refractory hypotension persisted. In such a situation, a single dose of glucagon markedly improved hemodynamics. Therefore, this case report is the first to demonstrate the efficacy and necessity of glucagon for refractory anaphylactic shock in patients taking β-blockers regularly whose hemodynamics do not improve despite initial treatment with adrenaline according to international guidelines.

Few reports have demonstrated the efficacy of glucagon for refractory anaphylactic shock. Surprisingly, a review by the European Anaphylaxis Registry reported that glucagon was rarely administered for refractory anaphylactic shock [14]. Similarly, in a report by the Royal College of Anaesthetists of 266 patients with severe intraoperative anaphylaxis, glucagon was administered (65 min after onset) in only 1 patient (who died) [15]. The reason glucagon is not administered for refractory anaphylaxis could be that glucagon is not widely recognized as a treatment for anaphylaxis [14, 15]. From a life-saving perspective, the use of second-line drugs for adrenaline-resistant anaphylactic shock is recommended [14, 15], and this case report supports glucagon administration to improve hemodynamics in patients taking β-blockers regularly.

In conclusion, we encountered a case of severe adrenaline-resistant anaphylactic shock caused by sugammadex in an elderly woman taking a selective β-1 blocker, which responded well to glucagon. Glucagon may improve hemodynamics in severe adrenaline-resistant anaphylactic shock patients taking β-blockers; however, its efficacy must be further evaluated in more cases.

## Data Availability

Not applicable.

## Abbreviations

ABP: Arterial blood pressure
AS: Aortic stenosis
BAV: Balloon aortic valvuloplasty
bpm: Beats per minute
HR: Heart rate
ppm: Pulses per minute
TAVI: Transcatheter aortic valve implantation
